# 7-Methoxytacrine-Adamantylamine Heterodimers as Cholinesterase Inhibitors in Alzheimer’s Disease Treatment — Synthesis, Biological Evaluation and Molecular Modeling Studies

**DOI:** 10.3390/molecules18022397

**Published:** 2013-02-20

**Authors:** Katarina Spilovska, Jan Korabecny, Jan Kral, Anna Horova, Kamil Musilek, Ondrej Soukup, Lucie Drtinova, Zuzana Gazova, Katarina Siposova, Kamil Kuca

**Affiliations:** 1 Department of Toxicology, Trebesska 1575, Faculty of Military Health Sciences, University of Defence, 500 01 Hradec Kralove, Czech Republic; E-Mails: spilovska@pmfhk.cz (K.S.); korabecny.jan@gmail.com (J.K.); horova.a@entrum.cz (A.H.); kamil.musilek@uhk.cz (K.M.); luciedrtinova@centrum.cz (L.D.); 2 University Hospital, Biomedicinal Research Centre, Sokolska 581, 500 05 Hradec Kralove, Czech Republic; E-Mail: soukup.ondrej07@gmail.com (O.S.); 3 Department of Pharmaceutical Chemistry and Drug Control, Faculty of Pharmacy, Charles University, Heyrovskeho 1203, 500 05 Hradec Kralove, Czech Republic; E-Mail: kral.jan@email.cz; 4 Department of Chemistry, Faculty of Sciences, University of Hradec Kralove, Rokitanskeho 62, 500 03 Hradec Kralove, Czech Republic; 5 Department of Biophysics, Institute of Experimental Physics, Slovak Academy of Sciences, Watsonova 47, 040 01 Kosice, Slovakia; E-Mails: gazova@saske.sk (Z.G.); katkasiposova@gmail.com (K.S.); 6 Faculty of Sciences, Institute of Chemistry, P. J. Safarik University, Srobarova 2, 041 54 Kosice, Slovakia; 7 Centre of Advanced Studies, Faculty of Military Health Sciences, University of Defence, Trebesska 1575, 500 01 Hradec Kralove, Czech Republic; E-Mail: kucakam@pmfhk.cz

**Keywords:** 7-MEOTA, amantadine, inhibitor, Alzheimer’s disease, acetylcholinesterase, butyrylcholinesterase

## Abstract

A structural series of 7-MEOTA-adamantylamine thioureas was designed, synthesized and evaluated as inhibitors of human acetylcholinesterase (hAChE) and human butyrylcholinesterase (hBChE). The compounds were prepared based on the multi-target-directed ligand strategy with different linker lengths (n = 2–8) joining the well-known NMDA antagonist adamantine and the hAChE inhibitor 7-methoxytacrine (7-MEOTA). Based on *in silico* studies, these inhibitors proved dual binding site character capable of simultaneous interaction with the peripheral anionic site (PAS) of hAChE and the catalytic active site (CAS). Clearly, these structural derivatives exhibited very good inhibitory activity towards hBChE resulting in more selective inhibitors of this enzyme. The most potent cholinesterase inhibitor was found to be thiourea analogue **14** (with an IC_50_ value of 0.47 µM for hAChE and an IC_50_ value of 0.11 µM for hBChE, respectively). Molecule **14** is a suitable novel lead compound for further evaluation proving that the strategy of dual binding site inhibitors might be a promising direction for development of novel AD drugs.

## 1. Introduction

Alzheimer’s disease (AD) is the most common form of dementia. It is the fourth leading cause of mortality in the US alone [1]. In Asia, AD is the principal cause of dementia and accounts for 50–60% of all cases, lasting for about 3–20 years from diagnosis to death. It was first documented in 1906 when Alois Alzheimer, a German psychiatrist and neuropathologist, reported the curious case of one of his patients, who suffered from memory problems, speaking impairment and difficulty with comprehensive understanding [2]. AD is a neurodegenerative disorder that results in the progressive and irreversible cognitive impairment, memory loss, and decline in language [3,4,5]. Several diverse hallmarks, such as deposits of aberrant proteins (β-amyloid and τ-protein), oxidative stress, dyshomeostasis of biometals, and low levels of acetylcholine (ACh) appear to play significant roles in the pathophysiology of the disease [6,7]. Moreover, AD also represents an economic burden, which causes profound social problems to both society and families [8,9].

**Figure 1:**
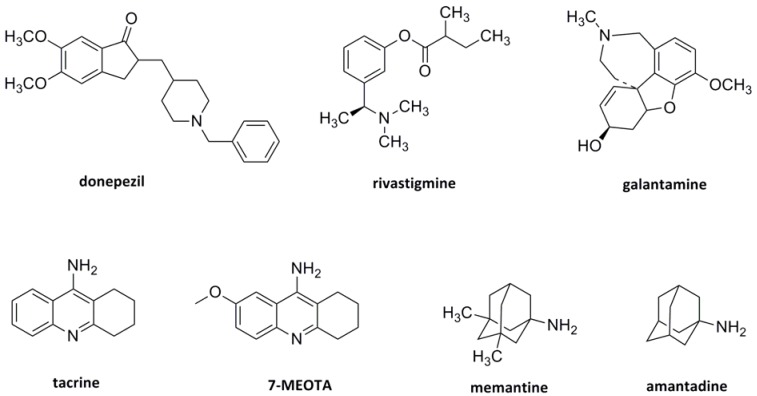
Structures of ChEIs, tacrine derivatives and polycyclic amines.

The current standard of care for mild to moderate AD, based on the so-called cholinergic hypothesis, includes treatment with cholinesterase inhibitors (ChEIs) to improve cognitive functions [5,10,11]. Several ChEIs have been approved by the US Food and Drug Administration (FDA), including tacrine, donepezil, rivastigmine and galantamine (Figure 1) [12,13,14,15,16]. Furthermore, memantine (1-amino-3,5-dimethyladamantane hydrochloride, Figure 1), an uncompetitive antagonist of *N*-methyl-D-aspartate (NMDA) receptors has been found to provide symptomatic benefits in clinical trials in AD patients [17,18,19]. Evidence of memantine’s neuroprotective effects (e.g., the ability to slow neurodegeneration) has been gathered primarily from preclinical models of the disease [20].

Memantine is a derivative of amantadine (1-adamantylamine hydrochloride, Figure 1), an antiviral agent that has long been used clinically to treat Parkinson’s disease (PD) in the US and in Europe. It also possesses antiepileptic properties, and is currently used in the AD treatment [21]. As an uncompetitive NMDA receptor antagonist, it proved low occurrence of side effects and showed good clinical tolerability in more than 200,000 treated patients [22]. It exhibited anti-neuroinflammatory effect with ability to act on glial cells. Memantine increased astroglial release of neurotrophic factors and inhibited inflammatory activation of microglia [23,24]. Amantadine is an established anti-PD agent with an inhibitory mechanism of action at NMDA receptors [25]. Indeed, evidence suggests that amantadine may delay the onset and severity of dementia related to PD similarly to its analogue memantine, which is regularly used in AD therapy [26,27]. 

In 1993, tacrine (9-amino-1,2,3,4-tetrahydroacridine, THA, Figure 1) was approved by the US Food and Drug Administration agency (FDA) as the first ChEI for the management of AD [28]. However, it was later withdrawn from the pharmaceutical market due to hepatotoxicity issues. The 7-methoxy derivative, 7-MEOTA (9-amino-7-methoxy-1,2,3,4-tetrahydroacridine, Figure 1) was found to be also an active ChEI with significantly lower side effects compared to THA [29,30,31,32,33,34].

Considering the complexity of AD, the standard, one molecule-one target solution may not be effective enough [35,36]. The novel “multi-target-directed strategy” has received attention, since a single molecule simultaneously interacts with multiple targets in the complex neuronal cascades. The multi-targeted analogues could achieve better efficacy by a complementary manner [36,37]. The studies following this strategy led to the synthesis of several chemically diverse structures with dual or multiple biological profiles [38], including acetylcholinesterase (AChE) and monoamine oxidase B (MAO-B) dual inhibitors [39,40,41], AChE and serotonin transporter (SER) dual inhibitor [42,43] and AChE, BACE, Aβ aggregation inhibiting and antioxidant multiple functional agents [44,45,46,47,48,49].

**Figure 2:**
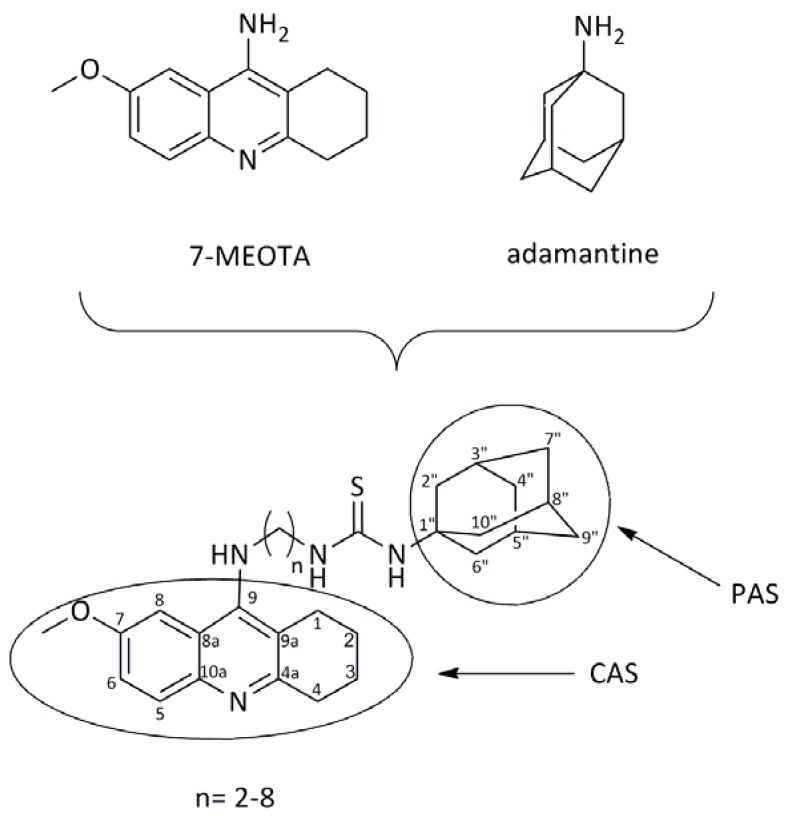
Design strategy of novel dual inhibitors.

In this presented work, the attention was focused on dual-binding site heterodimers. A series of 7-MEOTA-adamantylamine thioureas designed to simultaneously interact with the active and peripheral binding sites of both human AChE (hAChE, EC 3.1.1.7) and human butyrylcholinesterase (hBChE, EC 3.1.1.8) were synthesized. Novel analogues could be effective in the AD treatment with respect to their ability to interact with multiple targets. The synthesis, biological evaluation and molecular modeling studies of the new 7-MEOTA-adamantylamine thioureas is reported. The design strategy of 7-MEOTA-amantadine heterodimers is displayed in Figure 2.

## 2. Results and Discussion

**Scheme 1:**
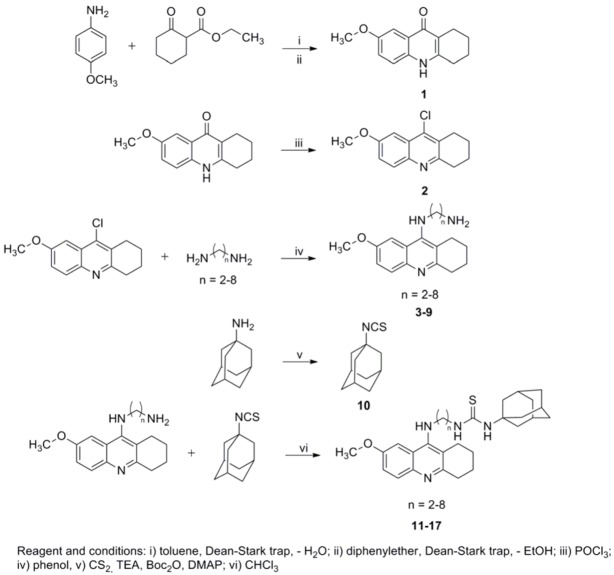
Synthetic route for preparation 7-MEOTA-adamantylamine thioureas **11**–**17**.

The general synthetic procedure for 7-MEOTA-adamantylamine heterodimers is shown in Scheme 1. The starting fused ring 7-methoxy-1,2,3,4-tetrahydroacridin-10*H*-9-one (**1**) was prepared in good yield (80%) by a condensation reaction of 4-methoxyaniline with ethyl 2-oxocyclohexanecarboxylate in refluxing toluene using addition of catalytic *p*-toluenesulfonic acid. Then, **1** was stirred with phosphorus oxychloride to give 9-chloro-7-methoxy-1,2,3,4-tetrahydroacridine (**2**) in quantitative yield. Spectral data were in good agreement with the literature characterization [28]. The treatment of **2** with appropriate 1,ω-diamines in the presence of phenol yielded the desired *N*-(7-methoxy-1,2,3,4-tetrahydroacridin-9-yl)alkane-1,ω-diamine intermediates **3**–**9** (70–95%).

1-Adamantyl isothiocyanate (**10**) was prepared in quantitative yield from 1-adamantyl amine via desulfurylated dithiocarbamate using carbon disulfide and di-*tert*-butyl dicarbonate. Spectral data were in good agreement with the literature [50,51,52].

Two synthons **10** and diamines **3**–**9** were utilized for formation of desired 7-MEOTA-adamantylamine thioureas **11**–**17**. All targeted compounds **11**–**17** were converted into tartaric salts to increase solubility. The final compounds were obtained as white-yellow powders in satisfactory yield (41–84%). Structural determination and signal assignments of thioureas were accomplished by the application of the usual combination of ^1^H- and ^13^C-NMR spectra.

**Figure 3:**
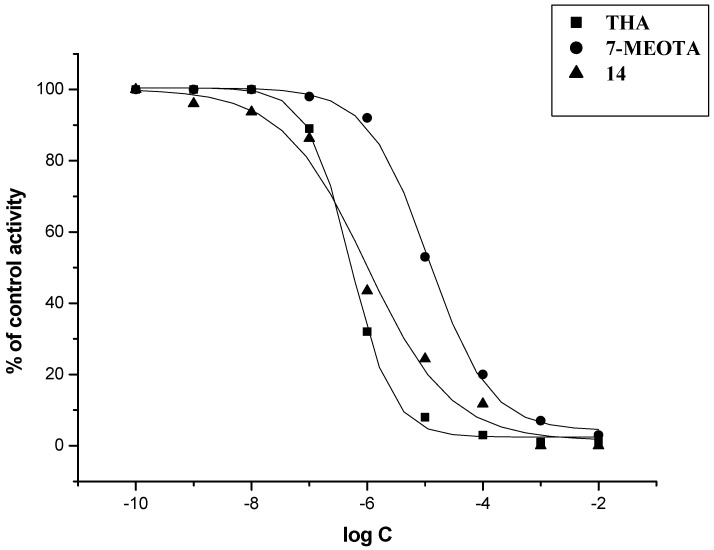
Enzyme activity plot of THA, 7-MEOTA and **14** for hAChE.

**Figure 4:**
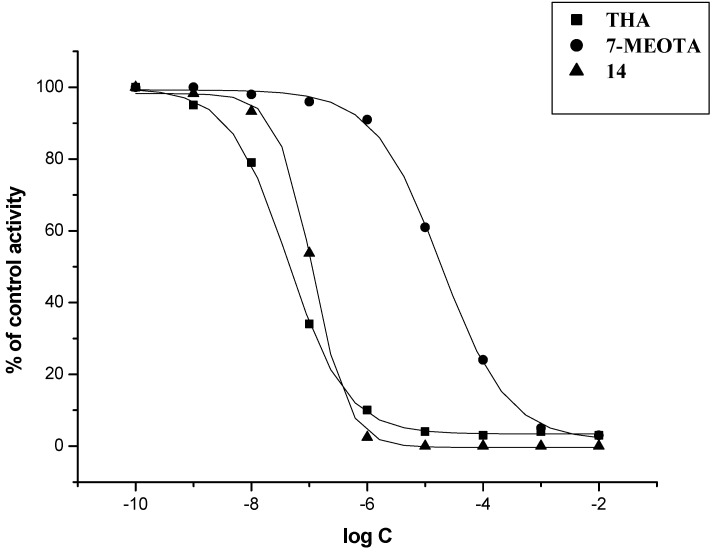
Enzyme activity plot of THA, 7-MEOTA and **14** for hBChE.

The *in vitro* activity of new 7-MEOTA-diamines **3**–**9** and 7-MEOTA-amantadine heterodimers **11**–**17** was determined on the model of human recombinant AChE (hAChE; E.C. 3.1.1.7) and human plasmatic BChE (hBChE; E.C. 3.1.1.8) using Ellman’s method [53,54] with THA, 7-MEOTA, amantadine and **10** as reference compounds (Table 1). THA was a more potent inhibitor of hAChE and hBChE in comparison to 7-MEOTA, amantadine and **10**. THA is a two orders of magnitude better inhibitor of hAChE than 7-MEOTA and **10** and a one order of magnitude better inhibitor of hAChE in comparison to amantadine. Additionally, 7-MEOTA, amantadine and **10** were weaker hBChE inhibitors compared to THA and showed higher selectivity. All intermediate diamines **3**–**9** were potent inhibitors of both cholinesterases, producing hAChE IC_50_ values in the range of 5.32–0.21 µM. Inhibitory activity of hBChE was less influenced by intermediate diamines **3**–**9** ranging from 64.64–7.26 µM. New prepared 7-MEOTA-diamines **3**–**9** did not exceed THA inhibitory activity toward hBChE, but diamine **7** showed higher inhibitory activity to hAChE. Moreover, all amines **3**–**9** are more potent inhibitors of hAChE than 7-MEOTA. From the series of 7-MEOTA-diamines, the best IC_50_ value was shown by diamine **7** bearing six carbons in the spacer. The selectivity index (SI, determined as the IC_50_hBChE/IC_50_hAChE ratio) was calculated for all newly evaluated compounds. The intermediates amines **3**–**9** displayed higher selectivity for hAChE than THA, 7-MEOTA, amantadine or **10**, while compound **7** expressed the highest selectivity index for hAChE. The amines were used as syntons for the synthesis of 7-MEOTA-amantadine heterodimers **11**–**17**. Surprisingly, the best inhibitory activity of thioureas **11**–**17** was demonstrated in thiourea analogue **14** with five carbons in the linker. Considering standards, inhibition activities towards both tested enzymes were exceeded only by compound **14**. Interestingly, compound **7** with six methylenes displayed a better IC_50_ value than derivative **14**, which had only five carbons between 7-MEOTA and adamantine moieties in the spacer (not counting the thiourea group). The novel thiourea derivatives proved to be better inhibitors of both cholinesterases compared with 7-MEOTA. However, some of the heterodimers exhibited slightly poorer hAChE and hBChE inhibitory activities in comparison with THA. The IC_50_ values suggested that compound **14** exerted similar IC_50_ in sub-µM range for hAChE/hBChE to reference compound THA. All of the novel compounds have lower SI values compared to 7-MEOTA, amantadine and **10**, so they can be considered as more selective agents for hBChE. This could provide an advantage for AD course as hBChE inhibition has recently been regarded therapeutically beneficial for the treatment of AD. Concentration of hBChE, contrary to hAChE, increases during the course of the AD and may compensate the role of hAChE [55]. Enzyme activity plots of THA, 7-MEOTA and **14** are displayed (Figure 3 and Figure 4). Hence, molecule **14** with the length of five methylene units in the linker remains the most potent hAChE/hBChE inhibitor among all the newly synthesized thioureas.

**Table 1:** IC_50_ values of amines, standards and tested thioureas.

Compound	IC_50_ (µM) ± SD ^b^		SI ^c^
	h AChE	hBChE	
THA	0.5 ± 0.1	0.023 ± 0.003	0.05
7-MEOTA	10.50 ± 2.40	21.0 ± 3.4	2.0
amantadine	16.05 ± 3.13	102.60 ± 17.13	6.4
3 ^a^	5.32 ± 1.04	64.45 ± 10.76	12.1
4 ^a^	1.93 ± 0.38	49.77 ± 8.31	25.8
5 ^a^	1.42 ± 0.28	9.22 ± 1.54	6.5
6 ^a^	3.44 ± 0.67	29.63 ± 4.95	8.6
7 ^a^	0.21 ± 0.04	10.84 ± 1.81	51.6
8 ^a^	0.86 ± 0.17	7.26 ± 1.21	8.4
9 ^a^	0.47 ± 0.09	10.08 ± 1.68	21.4
10	24.96 ± 4.87	96.90 ± 16.18	3.9
11	5.02 ± 0.98	6.02 ± 1.01	1.2
12	0.53 ± 0.10	1.39 ± 0.23	2.6
13	2.04 ± 0.39	0.98 ± 0.16	0.5
14	0.47 ± 0.09	0.11 ± 0.02	0.2
15	2.09 ± 0.40	0.33 ± 0.05	0.2
16	3.47 ± 0.67	0.15 ± 0.02	0.04
17	1.62 ± 0.31	0.26 ± 0.04	0.2

^a^ Compounds **3**–**9** were tested as dihydrochloride salts. ^b^ The *in vitro* concentration of tested compound required to produce 50% inhibition of hAChE or hBChE. Results are the mean of three independent determinations ± standard deviation. ^c^ Selectivity index (SI) for hAChE is determined as ratio of IC_50_ hBChE towards IC_50_ hAChE.

**Figure 5:**
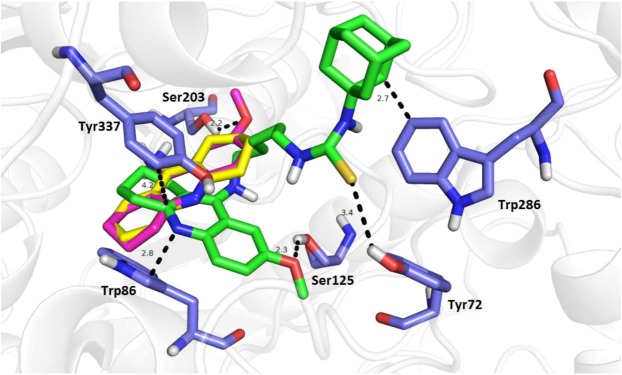
Putative binding mode of **14** (green), THA (yellow) and 7-MEOTA (magenta). Some important amino acid residues (blue) are shown, the rest of hAChE is superimposed in cartoon conformation.

**Figure 6:**
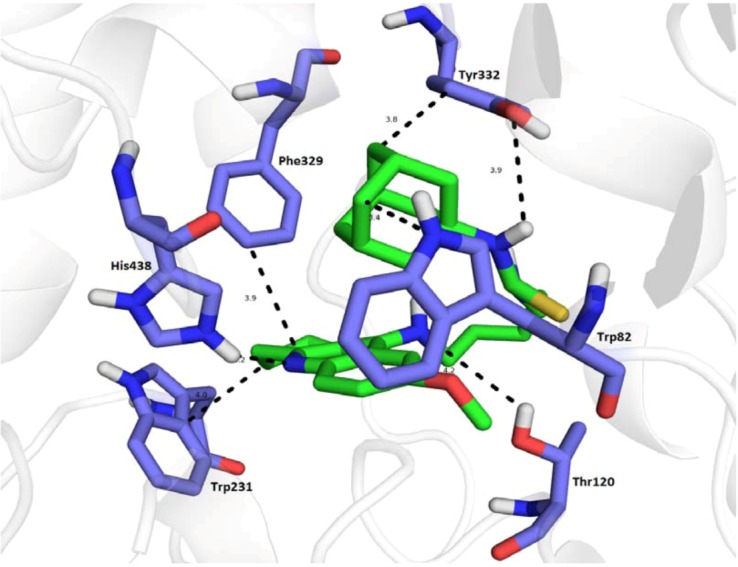
Docked complex of **14** (green) in the hBChE active site. Interaction with key amino acid residues (blue) are highlighted by black dashed lines. The rest of hBChE is illustrated as cartoon for clarity.

**Figure 7:**
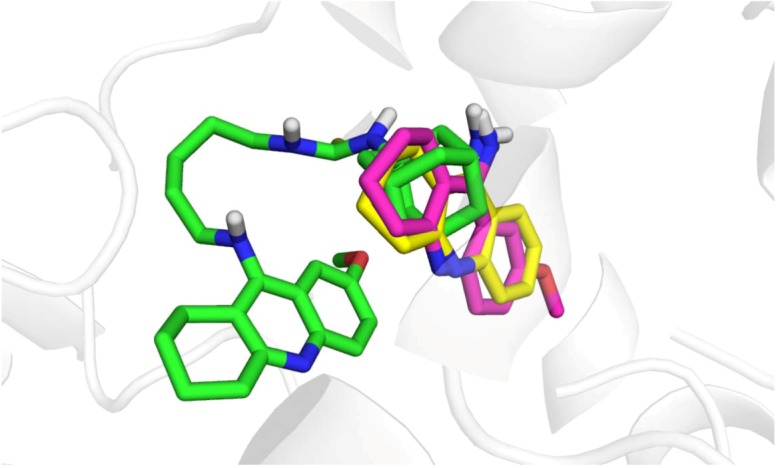
Top-scored docking poses representation within hBChE active site for **14** (green), THA (yellow) and 7-MEOTA (magenta).

To shed light onto the putative orientation of the most promising cholinesterase inhibitor **14** in both cholinesterases, molecular modeling studies were carried out using the Autodock Vina software. The molecular modelling results are shown in Figure 5, Figure 6, Figure 7. A structure of hAChE complexed with fasciculin (PDB ID: 1B41) was used for the *in silico* studies and exploited in the *in vitro* biochemical assays presented above. The proposed binding mode of **14**, THA and 7-MEOTA with the interacting key amino acid residues (in blue) in hAChE active site is shown in Figure 5. In complex with THA (−9.9 kcal/mol; in yellow), the three-ring structure is stacked against the phenyl ring of Tyr337 (3.4 Å) as well as showing T-shaped π-π bonding with Trp86 (1.0 Å). THA amino moiety is stabilized by hydrogen bond to carbonyl group of His447 (4.3 Å). Phe338 (4.3 Å) is weakly involved into the direct aliphatic-π interaction. All these findings fully correspond with that of previously published for THA in the active site of *Torpedo californica* AChE (TcAChE) [56]. Similar spatial conformation was found for the top-scored docking pose of 7-MEOTA (−9.8 kcal/mol, in magenta). Interestingly, 7-MEOTA-hAChE complex is additionally stabilized with hydrogen bonds of the methoxy moiety and Ser203 (2.2 Å) and the methoxy group is also attached to Gly122 (2.4 Å). Moreover, 7-MEOTA resulted 180 degrees rotated from THA with the same position of 9-amino moiety. Despite the fact of forming additional hydrogen bonds with the methoxy group of 7-MEOTA, this different spatial conformation compared to THA might explain its lower inhibition properties *in vitro*.

The binding mode for **14** (−11.1 kcal/mol, in green) suggested that the 7-MEOTA fragment was bound to near the bottom of the gorge in a slightly different arrangement compared to the parent compounds THA and 7-MEOTA. The 7-MEOTA scaffold of **14** exerted strong parallel π-π stacking to Tyr337 (4.2 Å) as well as T-shaped π-π stacking to Trp86 (2.8 Å) within the cation-π site of hAChE. The methoxy moiety contributed to stabilization of **14**-hAChE complex by hydrogen bonding with Ser125 (2.3 Å). The linkers showed moderate importance on cholinesterase inhibition as the analogue **14** emerged as the most promising with the length of five methylenes between 7-MEOTA scaffold and adamantyl moiety with small difference to other thioureas. Similar results can be observed for different series of multi-target directed ligands based on tacrine scaffold including bis-7-tacrine, tacrine-piperazine derivatives, tacrine-hupyridone dimers, tacrine-melatonin heterodimers, pyrano[3,2-c]-quinoline-6-chlorotacrine hybrids and tacrine-ferulic acid nitric-oxide donors [45,47,57,58,59,60]. In the middle of the gorge, the aliphatic alkyl chain of **14** was surrounded with phenyl rings of Tyr124 (3.7 Å), Phe297 (3.5 Å) and Phe338 (4.3 Å) providing **14**-hAChE complex stabilization and constriction. Thiourea group forms very weak hydrogen bonding to hydroxyl group of Tyr72 (3.4 Å). At the rim of the gorge within peripheral anionic site, adamantyl moiety may have aliphatic-π contact with Trp286 (2.7 Å) and several weak van der Waals interactions (e.g., Leu289—3.3 Å, Ser293—3.7 Å). Lower affinity of **14** for hAChE inhibition might be explained by non-aromatic character of interactions with adamatyl moiety between the key residues in peripheral anionic site. Finally, the distance between 7-MEOTA and adamantyl moieties had a length of 18.2 Å which correlates with the ideas of dual binding site heterodimers, where the distance between peripheral anionic site and catalytic site of hAChE is estimated to about 20 Å [61].

The crystal structure of THA with hBChE is not available for molecular modeling. To gain insight into the molecular determinants that modulate the inhibitory activity of the novel 7-MEOTA-adamantine hybrids, the crystal structure of hBChE (PDB ID: 1P01) that was previously modeled with bound butyrate was chosen. Flexible docking studies for hBChE showed different spatial orientation for standard compounds (THA, 7-MEOTA) and **14** (Figure 6 and Figure 7). Focused on reference compounds, THA (−9.5 kcal/mol, in yellow) and 7-MEOTA (−9.3 kcal/mol, in magenta) showed very similar orientation with several apparent π-π interactions, that were for THA mainly in T-shaped orientation (Tyr332—3.3 Å, Trp430—4.6 Å, Trp82—2.4 Å) as well as parallel oriented (Tyr440—3.7 Å). Hydrogen bonds are formed between THA amino group and Trp430 (3.7 Å) and Ser79 (3.6 Å). Interestingly, 7-MEOTA provided almost identical putative orientation with additional hydrogen bond of Trp82 (3.5 Å) towards the methoxy moiety. According to *in silico* results, 7-MEOTA could be considered a stronger hBChE inhibitor than THA, but the obtained *in vitro* results highlighted THA as a three orders of magnitude better BChE inhibitor. The three-ring core of analogue **14** (−10.3 kcal/mol, in green) establishes π-π interactions with Trp231 (4.0 Å) and Phe329 (3.9 Å). The nitrogen atom of the tacrine moiety is bridged to His438 via hydrogen bonding (2.2 Å), the secondary amino group preserves a weak H-bond to the hydroxyl group of Thr120 (4.2 Å). The dual binding character of Tyr332 is depicted. This amino acid residue allows H-bonds with its OH moiety to the thiourea linker (3.9 Å). Aliphatic-π contact can be observed within adamantyl skeleton and Tyr332 (3.8 Å) as well as with Trp82 (3.4 Å). Finally, the *in silico* calculated binding energies for hBChE suggested similar or even higher affinity towards this enzyme, but *in vitro* **14** resulted one order of magnitude weaker inhibitor of hBChE reflected to THA.

Structural determination and signal assignment of thioureas **11**–**17** were accomplished by the application of the usual combination of ^1^H and ^13^C-NMR spectra. The unequivocal assignments were performed by homo- and hetero-correlated two-dimensional NMR experiments (H,H-COSY, H,C-HSQC, H,C-HMBC). Infrared spectroscopy was conducted only for **10** to observe the isothiocyanate group vibrations. The ^1^H- and ^13^C-NMR spectra, synthesis analyses and mass spectra are collected in the Experimental section. For the aromatic part of 7-MEOTA moiety correlations between signals at 7.74–7.79 ppm and 7.35–7.47 ppm allow the assignment of H-5 and H-6, respectively. The signal at 7.53–7.63 ppm is assigned to H-8. For the alicyclic part of 7-MEOTA skeleton correlations between signals of the multiplet at 1.79–1.82 ppm allow the assignment of H-2 and H-3, and H-1 and H-4 were assigned based on the correlation between the triplets at 2.69–2.76 ppm and 2.93–2.97 ppm, respectively. The OCH_3_ carbon signal was conclusively assigned on the basis of the correlation between 3.88–3.91 ppm. As for the adamantyl-moiety correlation between the multiplet at 1.53–2.00 allow the assignment of H-4′′, H-7′′, H-9′′ to be unequivocally assigned. For the part of the adamantyl-moiety correlation at 1.97–2.00 allows assignment of H-3′′, H-5′′, H-8′′. The multiplet at 2.05–2.13 is assigned to H-2′′, H-6′′, H-10′′. Thioureas **11**–**17** displayed a typical C=S carbon resonance at 180.8–181.1 ppm. The thioureas **11**–**17** were each converted into the thioureas dihydroxysuccinates using tartaric acid. These thiourea dihydroxysuccinates were determined on the correlation between the multiplet at 3.98–4.09 ppm to H-2′′′, H-3′′′ and the carbon signal of C-1′′′, C-4′′′ allowing assignment of signals at 174.0–174.6 ppm, and 71.8–72.0 ppm to C-2′′′, C-3′′′, respectively.

## 3. Experimental

### 3.1. Chemistry

7-MEOTA was prepared at our department according to the method described earlier [15]. All reagents were reagent grade quality and obtained from Sigma-Aldrich (Prague, Czech Republic). All experiments were carried out under nitrogen atmospheres. Thin layer chromatography (TLC) was performed on aluminium sheets precoated with silica gel 60 F254 (Merck, Prague, Czech Republic). Column chromatography was performed at normal pressure on silica gel 100 (particle size 0.063–0.200 mm, 70–230 mesh ASTM, Fluka, Prague, Czech Republic). Elemental analysis was measured at Perkin-Elmer CHN Analyser 2,400 Serie II apparatus. Mass spectra were recorded using a combination of high performance liquid chromatography and mass spectrometry. The HP1100 HPLC system was obtained from Agilent Technologies (Waldbronn, Germany). It consisted of a G1322A vacuum degasser, G1311A quaternary pump, G1313A autosampler and a MSD1456 VL quadrupole mass spectrometer equipped with an electrospray ionization source. Nitrogen for mass spectrometer was supplied by a Whatman 75–720 nitrogen generator. Data were collected in positive ion mode with an ESI probe voltage of 4000 V. The pressure of nebulizer gas was set up to 35 psig. Drying gas temperature was operated at 335 °C and flow at 13 L/min. ^1^H-NMR and ^13^C-NMR spectra were recorded with a Varian S500 spectrometer operating at 500 and 125 MHz, respectively, in deuteriochloroform (CDCl_3_; 7.27 (D), 77.2 (C) ppm) or hexadeuteriodimethylsulfoxide (DMSO-*d_6_*; 2.50 (D), 39.7 (C) ppm) using tetramethylsilane (TMS) as internal reference (=0 ppm for both nuclei). Chemical shifts are reported in parts per milion (ppm, δ) relative to TMS. The assignment of chemical shifts is based on standard NMR experiments (^1^H, ^13^C, ^1^H-^1^H COSY, ^1^H-^13^C HSQC, HMBC, DEPT). Melting points were measured on a micro heating stage PHMK 05 (VEB Kombinant Nagema, Radebeul, Germany) and are uncorrected. 

### 3.2. *In Vitro* Evaluation

A Sunrise multichannel spectrophotometer (Tecan, Salzburg, Austria) was used for all cholinesterase activity measurements. A previously optimized Ellman procedure was slightly modified in order to estimate anticholinergic properties [53,54]. 96-well photometric microplates made from polystyrene (Nunc, Rockilde, Denmark) were used for measuring purposes. Human recombinant AChE or human plasmatic BChE (Aldrich; commercially purified by affinity chromatography) were suspended into phosphate buffer (pH 7.4) up to final activity 0.002 U/μL. Cholinesterase (5 μL), freshly mixed solution of 0.4 mg/mL 5,5′-dithio-bis(2-nitrobenzoic) acid (40 μL), 1 mM acetylthiocholine chloride in phosphate buffer (20 μL) and appropriate concentration of inhibitor (1 mM–0.1 nM; 5 μL) were injected per well. Absorbance was measured at 412 nm after 5 min incubation using automatic shaking of the microplate. The obtained data were used to compute percentage of inhibition [I; Equation (1)]:

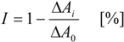
(1)
where ΔA_i_ indicates absorbance change provided by cholinesterase exposed to hAChE inhibitors and ΔA_0_ indicates absorbance change caused by intact cholinesterase (phosphate buffer was applied instead of hAChE inhibitor). IC_50_ values were calculated using Origin 6.1 (Northampton, MA, USA). Percentage of inhibition for the given anticholinergic compound was overlaid by proper curve chosen according to optimal correlation coefficient. IC_50_ as well as upper limit of inhibition (maximal inhibition provided by given compound) was computed.

### 3.3. General Procedure for Synthesis of N-(7-Methoxy-1,2,3,4-tetrahydroacridin-9-yl)alkane-1,ω-diamines *(**3–9**)*

Phenol (10.0 g) and **2** (0.5 g, 2.0 mmol) was heated and stirred at 80–90 °C until a homogenous solution was obtained. The appropriate 1,ω-diaminoalkane (0.49 g, 8.1 mmol) was added and the temperature of the reaction was raised to 125–130 °C and maintained until starting material **2** disappeared (2–4 h). After cooling the mixture was poured into 20% sodium hydroxide and extracted with dichloromethane. The organic layer was washed with brine and water, dried over sodium sulphate and evaporated to dryness under reduced pressure. The oily residue was purified via flash chromatography EtOAc/MeOH/NH_3_ (25% aq.) (6:2:0.2) as eluent to give intermediates **3**–**9**.

*N-(7-Methoxy-1,2,3,4-tetrahydroacridin-9-yl)ethane-1,2-diamine* (**3**). Yellow oil, yield: 90%; ^1^H-NMR (CDCl_3_) *δ* 1.91 (m, 4H, 2 × CH_2_, H-2, H-3), 2.80 (m, 2H, CH_2_, H-1), 2.95 (t, 2H, CH_2_, H-2′, *J* = 5.6 Hz), 3.04 (m, 2H, CH_2_, H-4), 3.43 (t, 2H, CH_2_, H-1′, *J* = 5.6 Hz), 3.91 (s, 3H, OCH_3_), 7.25(ddd, 1H, CH, H-6, *J* = 9.2, 6.0, 2.4 Hz), 7.32 (d, 1H, CH, H-8, *J* = 2.8 Hz), 7.84 (d, 1H, CH, H-5, *J* = 9.2 Hz); ^13^C-NMR (CDCl_3_) *δ* 22.9, 23.1 (C-2, C-3), 24.9 (C-1), 33.7 (C-4), 42.4 (C-2′), 50.8 (C-1′), 55.5 (OCH_3_), 101.7 (C-8), 117.9 (C-9a), 120.3 (C-6), 121.5 (C-8a), 130.2 (C-5), 143.2 (C-10a), 150.0 (C-9), 156.0 (C-7), 156.2 (C-4a); Elemental analysis: calculated 70.82% C, 7.80% H, 15.49% N; found 70.99% C, 6.55% H, 15.60% N; ESI-MS: *m/z* 270.1 [M]^+^ (calculated for: [C_16_H_22_N_3_O]^+^ 270.2).

*N-(7-Methoxy-1,2,3,4-tetrahydroacridin-9-yl)propane-1,3-diamine* (**4**). Yellow oil, yield: 88%; ^1^H-NMR (CDCl_3_) *δ* 1.82 (m, 2H, CH_2_, H-2′, *J* = 6.4 Hz), 1.89 (m, 4H, 2 × CH_2_, H-2, H-3), 2.74 (m, 2H, CH_2_, H-1), 2.92 (m, 2H, CH_2_, H-3′, *J* = 6.8 Hz), 3.04 (m, 2H, CH_2_, H-4), 3.55 (t, 2H, CH_2_, H-1′, *J* = 6.4 Hz), 3.90 (s, 3H, OCH_3_), 5.44 (bs, 1H, NH), 7.23 (dd, 1H, CH, H-6, *J* = 9.2, 2.8 Hz), 7.28 (d, 1H, CH, H-8, *J* = 2.8 Hz), 7.85 (d, 1H, CH, H-5, *J* = 9.2 Hz); ^13^C-NMR (CDCl_3_) *δ* 22.6, 22.9 (C-2, C-3), 25.0 (C-1), 33.2 (C-2′), 33.8 (C-4), 40.4 (C-3′), 47.6 (C-1′), 55.4 (OCH_3_), 101.8 (C-8), 116.8 (C-9a), 120.4 (C-6), 120.9 (C-8a), 129.4 (C-5), 142.5 (C-10a), 150.3 (C-9), 155.7 (C-7), 155.9 (C-4a); Elemental analysis: calculated 71.55% C, 8.12% H, 14.72% N; found 71.85% C, 8.25% H, 14.85% N; ESI-MS: *m/z* 286.2 [M]^+^ (calculated for: [C_17_H_24_N_3_O]^+^ 286.2).

*N-(7-Methoxy-1,2,3,4-tetrahydroacridin-9-yl)butane-1,4-diamine* (**5**). Yellow oil, yield: 85%; ^1^H-NMR (CDCl_3_) *δ* 1.57 (m, 2H, CH_2_, H-3′), 1.71 (m, 2H, CH_2_, H-2′), 1.90 (m, 4H, 2 × CH_2_, H-2, H-3), 2.74 (m, 4H, 2 × CH_2_, H-1,4′), 3.03 (m, 2H, CH_2_, H-4), 3.43 (t, 2H, CH_2_, H-1′, *J* = 6.4 Hz), 3.91 (s, 3H, OCH_3_), 5.75 (bs, 1H, NH), 7.24 (m, 2H, 2 × CH, H-6, H-8), 7.83 (d, 1H, CH, H-5, *J* = 9.2 Hz); ^13^C-NMR (CDCl_3_) *δ* 22.8, 23.1 (C-2, C-3), 24.8 (C-1), 29.1 (C-2′), 30.8 (C-3′), 33.6 (C-4), 41.7 (C-4′), 48.9 (C-1′), 55.5 (OCH_3_), 101.7 (C-8), 117.2 (C-9a), 120.4 (C-6), 121.2 (C-8a), 130.0 (C-5), 143.0 (C-10a), 150.0 (C-9), 156.0 (C-7,4a); Elemental analysis: calculated 72.21% C, 8.42% H, 14.03% N; found 72.15% C, 8.19% H, 14.35% N; ESI-MS: *m/z* 300.2 [M]^+^ (calculated for: [C_18_H_26_N_3_O]^+^ 300.2).

*N-(7-Methoxy-1,2,3,4-tetrahydroacridin-9-yl)pentane-1,5-diamine* (**6**). Yellow oil, yield: 93%; ^1^H-NMR (CDCl_3_) *δ* 1.47 (m, 4H, 2 × CH_2_, H-3′, H-4′), 1.68 (m, 2H, CH_2_, H-2′), 1.91 (m, 4H, 2 × CH_2_, H-2, H-3), 2.71 (m, 4H, 2 × CH_2_, H-1, H-5′), 3.04 (m, 2H, CH_2_, H-4), 3.41 (t, 2H, CH_2_, H-1′, *J* = 6.4 Hz), 3.91 (s, 3H, OCH_3_), 7.23 (m, 2H, 2 × CH, H-6, H-8 Hz), 7.83 (d, 1H, CH, H-5, *J* = 9.2 Hz); ^13^C-NMR (CDCl_3_) *δ* 22.8, 23.1 (C-2, C-3), 24.4 (C-3′), 24.8 (C-1), 31.7 (C-2′), 33.5 (C-4′), 33.8 (C-4), 42.0 (C-5′), 49.1 (C-1′), 55.5 (OCH_3_), 101.7 (C-8), 117.4 (C-9a), 120.3 (C-6), 121.3 (C-8a), 130.3 (C-5), 143.4 (C-10a), 149.8 (C-9), 155.9 (C-7), 156.2 (C-4a); Elemental analysis: calculated 72.81% C, 8.68% H, 13.41% N; found 72.5% C, 8.35% H, 13.62% N; ESI-MS: *m/z* 314.2 [M]^+^ (calculated for: [C_19_H_28_N_3_O]^+^ 314.2).

*N-(7-Methoxy-1,2,3,4-tetrahydroacridin-9-yl)hexane-1,6-diamine* (**7**). Yellow oil, yield: 81%; ^1^H-NMR (CDCl_3_) *δ* 1.41 (m, 6H, 3 × CH_2_, H-3′, H-4′, H-5′), 1.67 (m, 2H, CH_2_, H-2′), 1.91 (m, 4H, 2 × CH_2_, H-2, H-3), 2.68 (t, 2H, CH_2_, H-6′, *J* = 7.2 Hz), 2.73 (m, 2H, CH_2_, H-1), 3.04 (m, 2H, CH_2_, H-4), 3.41 (t, 2H, CH_2_, H-1′, *J* = 7.2 Hz), 3.90 (s, 3H, OCH_3_), 7.24 (m, 2H, 2 × CH, H-6, H-8), 7.84 (d, 1H, CH, H-5, *J* = 9.2 Hz); ^13^C-NMR (CDCl_3_) *δ* 22.8, 23.1 (C-2, C-3), 24.7 (C-1), 26.7, 26.9 (C-3′, C-4′), 31.8 (C-2′), 33.4 (C-5′), 33.7 (C-4), 42.0 (C-6′), 49.1 (C-1′), 55.5 (OCH_3_), 101.7 (C-8), 117.2 (C-9a), 120.3 (C-6), 121.2 (C-8a), 130.1 (C-5), 143.2 (C-10a), 150.0 (C-9), 155.9 (C-7), 156.1 (C-4a); Elemental analysis: calculated 73.36% C, 8.93% H, 12.83% N; found 73.51% C, 8.68% H, 12.85% N; ESI-MS: *m/z* 328.2 [M]^+^ (calculated for: [C_20_H_30_N_3_O]^+^ 328.2).

*N-(7-Methoxy-1,2,3,4-tetrahydroacridin-9-yl)heptane-1,7-diamine* (**8**). Yellow oil, yield: 91%; ^1^H-NMR (CDCl_3_) *δ* 1.32 (m, 6H, 3 × CH_2_, H-3′, H- 4′, H-5′), 1.42 (m, 2H, CH_2_, H-6′), 1.66 (m, 2H, CH_2_, H-2′), 1.91 (m, 4H, 2 × CH_2_, H-2, H-3), 2.67 (t, 2H, CH_2_, H-7′, *J* = 7.2 Hz), 2.73 (m, 2H, CH_2_, H-1), 3.04 (m, 2H, CH_2_, H-4), 3.40 (t, 2H, CH_2_, H-1′, *J* = 7.2 Hz), 3.91 (s, 3H, OCH_3_), 7.25 (m, 2H, 2 × CH, H-6, H-8), 7.84 (d, 1H, CH, H-5, *J* = 9.2 Hz); ^13^C-NMR (CDCl_3_) *δ* 22.8, 23.1 (C-2, C-3), 24.7 (C-1), 26.8, 27.0, 29.3 (C-3′, C-4′, C-5′), 31.7 (C-2′), 33.4 (C-6′), 33.7 (C-4), 42.0 (C-7′), 49.2 (C-1′), 55.5 (OCH_3_), 101.7 (C-8), 117.2 (C-9a), 120.3 (C-6), 121.2 (C-8a), 130.1 (C-5), 143.2 (C-10a), 150.0 (C-9), 155.9 (C-7), 156.1 (C-4a); Elemental analysis: calculated 73.86% C, 9.15% H, 12.30% N; found 73.75% C, 9.10% H, 12.45% N; ESI-MS: *m/z* 342.2 [M]^+^ (calculated for: [C_21_H_32_N_3_O]^+^ 342.3).

*N-(7-Methoxy-1,2,3,4-tetrahydroacridin-9-yl)octane-1,8-diamine* (**9**). Yellow oil, yield: 84%; ^1^H-NMR (CDCl_3_) *δ* 1.35 (m, 6H, 3 ×CH_2_, H-3′, H-4′, H-5′), 1.51 (m, 2H, CH_2_, H-6′), 1.73 (m, 2H, CH_2_, H-2′), 1.91 (m, 4H, 2 × CH_2_, H-2, H-3), 2.50 (m, 2H, CH_2_, H-7′), 2.62 (t, 2H, CH_2_, H-8′, *J* = 7.0 Hz), 2.73 (m, 2H, CH_2_, H-1), 3.04 (m, 2H, CH_2_, H-4), 3.42 (t, 2H, CH_2_, H-1′, *J* = 7.2 Hz), 3.89 (s, 3H, OCH_3_), 7.27 (m, 2H, 2 × CH, H-6, H-8), 7.80 (d, 1H, CH, H-5, *J* = 9.2 Hz); ^13^C-NMR (CDCl_3_) *δ* 22.9, 23.4 (C-2, C-3), 24.7 (C-1), 26.9, 27.0, 29.4 (C-3′, C-4′, C-5′), 31.5 (C-2′), 33.6 (C-6′), 33.8 (C-4), 42.0 (C-7′), 43.2 (C-8′), 49.2 (C-1′), 55.9 (OCH_3_), 101.6 (C-8), 116.9 (C-9a), 120.1 (C-6), 121.2 (C-8a), 130.5 (C-5), 141.0 (C-10a), 150.8 (C-9), 155.9 (C-7), 156.0 (C-4a); Elemental analysis: calculated 74.32% C, 9.36% H, 11.82% N; found 74.38% C, 9.15% H, 12.00% N; ESI-MS: *m/z* 356.2 [M]^+^ (calculated for: [C_22_H_34_N_3_O]^+ ^356.3).

### 3.4. General Procedure for Synthesis of 1-Adamantyl-3-(2-(7-methoxy-1,2,3,4-tetrahydroacridin-9-yl-amino)alkane)thiourea 2,3-dihydroxysuccinates ***11–17***

*N*-(7-methoxy-1,2,3,4-tetrahydroacridin-9-yl)alkane-1,ω-diamines (**3**–**9**) (10 mmol) and 1-adamantyl isothiocyanate (**10**, 12 mmol) were dissolved in CHCl_3_ and stirred 24 h at room temperature. The crude products were evaporated to dryness and purified via flash chromatography CHCl_3_/MeOH (9:1). Pure basis were converted to tartaric salts by addition of equimolar tartatic acid and stirred in absolute ethanol (10 ml) for 24 h. Thiourea dihydroxysuccinates **11**–**17** were obtained as white-yellow powders in good yields. 

*1-Adamantyl-3-(2-(7-methoxy-1,2,3,4-tetrahydroacridin-9-yl-amino)ethyl)thiourea 2,3-dihydroxysuccinate* (**11**). White-yellow powder, yield: 81.2%; m.p. = 90.1–93.2 °C; ^1^H-NMR (DMSO-*d_6_*) *δ* 1.53 (m, 6H, 3 × CH_2_, H-4′′, H-7′′, H-9′′), 1.79 (m, 4H, 2 × CH_2_, H-2, H-3), 1.97 (m, 3H, 3 × CH, H-3′′, H-5′′, H-8′′), 2.05 (m, 6H, 3 × CH_2_, H-2′′, H-6′′, H-10′′), 2.76 (t, 2H, CH_2_, H-1, *J* = 5.7 Hz), 2.95 (t, 2H, CH_2_, H-4, *J* = 6.0 Hz), 3.73 (m, 2H, CH_2_, H-2′′), 3.84 (m, 2H, CH_2_, H-1′′), 3.91 (s, 3H, OCH_3_), 4.09 (m, 2H, 2 × CH, H-2′′′, H-3′′′), 7.01 (bs, 1H, NH), 7.22 (bs, 1H, NH), 7.42 (dd, 1H, CH, H-6, *J* = 9.2, 2.4 Hz), 7.63 (m, 1H, CH, H-8), 7.78 (d, 1H, CH, H-5, *J* = 9.2 Hz); ^13^C-NMR (DMSO-*d_6_*) *δ* 21.1, 22.2 (C-2, C-3), 25.3 (C-1), 29.2 (C-3′′, C-5′′, C-8′′), 29.5 (C-4), 36.1 (C-4′′, C-7′′, C-9′′), 41.2 (C-2′′, C-6′′, C-10′′), 43.2 (C-2′), 47.6 (C-1′), 52.9 (C-1′′), 56.0 (OCH_3_), 72.0 (C-2′′′, C-3′′′), 103.2 (C-8), 113.3 (C-9a), 118.7 (C-8a), 123.0 (C-6), 123.6 (C-5), 135.5 (C-10a), 151.6 (C-4a), 153.9 (C-9), 156.4 (C-7), 174.1 (C-1′′′, C-4′′′),181.1 (C=S); Elemental analysis: calculated 60.57% C, 6.89% H, 9.11% N, 5.22% S; found 60.32% C, 6.83% H, 8.95% N, 5.20% S; ESI-MS: *m/z* 465.2 [M]^+^ (calculated for: [C_27_H_37_N_4_OS]^+^ 465.3).

*1-Adamantyl-3-(2-(7-methoxy-1,2,3,4-tetrahydroacridin-9-yl-amino)propyl)thiourea 2,3-dihydroxy-succinate* (**12**). White-yellow powder, yield: 41.0%; m.p. = 95.2–97.8 °C; ^1^H-NMR (DMSO-*d_6_*) *δ* 1.58 (m, 6H, 3 × CH_2_, H-4′′, H-7′′, H-9′′), 1.82 (m, 6H, 2 × CH_2_, H-2, H-3, H-2′), 1.99 (m, 3H, 3 × CH, H-3′′, H-5′′, H-8′′), 2.07 (m, 6H, 3 × CH_2_, H-2′′, H-6′′, H-10′′), 2.73 (m, 2H, CH_2_, H-1), 2.95 (t, 2H, CH_2_, H-4, *J* = 5.7 Hz), 3.44 (m, 2H, CH_2_, H-3′), 3.68 (t, 2H, CH_2_, H-1′, *J* = 6.3 Hz), 3.90 (s, 3H, OCH_3_), 4.07 (m, 2H, 2 × CH, H-2′′′, H-3′′′), 7.00 (bs, 1H, NH), 7.47 (dd, 1H, CH, H-6, *J* = 9.2, 2.4 Hz), 7.59 (m, 1H, CH, H-8), 7.77 (d, 1H, CH, H-5, *J* = 9.2 Hz); ^13^C-NMR (DMSO-*d_6_*) *δ* 21.2, 22.2 (C-2, C-3), 25.1 (C-1), 29.2 (C-3′′, C-5′′, C-8′′), 29.7 (C-4), 30.7 (C-3′), 36.1 (C-4′′, C-7′′, C-9′′), 40.2 (C-2′′, C-6′′, C-10′′), 41.3 (C-2′), 44.7 (C-1′), 52.8 (C-1′′), 56.0 (OCH_3_), 71.9 (C-2′′′, C-3′′′), 103.0 (C-8), 113.3 (C-9a), 118.7 (C-8a), 122.9 (C-6), 123.9 (C-5), 135.9 (C-10a), 151.7 (C-4a), 153.4 (C-9), 156.4 (C-7), 174.0 (C-1′′′, C-4′′′), 180.8 (C=S); Elemental analysis: calculated 61.13% C, 7.05% H, 8.91% N, 5.10% S; found 61.02% C, 6.98% H, 8.98% N, 5.13% S; ESI-MS: *m/z* 479.2 [M]^+^ (calculated for: [C_28_H_39_N_4_OS]^+^ 479.3).

*1-Adamantyl-3-(2-(7-methoxy-1,2,3,4-tetrahydroacridin-9-yl-amino)butyl)thiourea 2,3-dihydroxysuccinate* (**13**). White-yellow powder, yield: 69.2%; m.p. = 80.3–84.2 °C; ^1^H-NMR (DMSO-*d_6_*) *δ* 1.45 (m, 2H, CH_2_, H-3′), 1.58 (m, 8H, 4 × CH_2_, H-2′, H-4′′, H-7′′, H-9′′), 1.80 (m, 4H, 2 × CH_2_, H-2, H-3), 1.98 (m, 3H, 3 × CH, H-3′′, H-5′′, H-8′′), 2.08 (m, 6H, 3 × CH_2_, H-2′′, H-6′′, H-10′′), 2.71 (t, 2H, CH_2_, H-1, *J* = 5.8 Hz), 2.94 (t, 2H, CH_2_, H-4, *J* = 5.8 Hz), 3.33 (m, 2H, CH_2_, H-4′), 3.62 (t, 2H, CH_2_, H-1′, *J* = 6.9 Hz), 3.89 (s, 3H, OCH_3_), 4.02 (m, 2H, 2 × CH, H-2′′′, H-3′′′), 6.72 (bs, 1H, NH), 6.91 (bs, 1H, NH), 7.38 (dd, 1H, CH, H-6, *J* = 9.2, 2.4 Hz), 7.56 (m, 1H, CH, H-8), 7.76 (d, 1H, CH, H-5, *J* = 9.2 Hz); ^13^C-NMR (DMSO-*d_6_*) *δ* 21.4, 22.3 (C-2, C-3), 25.0 (C-1), 26.3 (C-4′), 28.2 (C-3′′, C-5′′, C-8′′), 29.2 (C-4), 30.0 (C-3′), 36.1 (C-4′′, C-7′′, C-9′′), 41.4 (C-2′′, C-6′′, C-10′′), 42.7 (C-2′), 47.0 (C-1′), 52.7 (C-1′′), 55.9 (OCH_3_), 71.8 (C-2′′′, C-3′′′), 102.9 (C-8), 113.7 (C-9a), 118.9 (C-8a), 122.5 (C-6), 124.6 (C-5), 136.7 (C-10a), 152.1 (C-4a), 153.0 (C-9), 156.2 (C-7), 174.2 (C-1′′′, C-4′′′), 180.8 (C=S); Elemental analysis: calculated 61.66% C, 7.21% H, 8.72% N, 4.99% S; found 61.30% C, 7.32% H, 8.60% N, 5.20% S; ESI-MS: *m/z* 493.2 [M]^+^ (calculated for: [C_29_H_41_N_4_OS]^+^ 493.3).

*1-Adamantyl-3-(2-(7-methoxy-1,2,3,4-tetrahydroacridin-9-yl-amino)pentyl)thiourea 2,3-dihydroxy-succinate* (**14**). White-yellow powder, yield: 54.1%; m.p. = 79.5–82.1 °C; ^1^H-NMR (DMSO-*d_6_*) *δ* 1.33 (m, 2H, CH_2_, H-4′), 1.43 (m, 2H, CH_2_, H-2′), 1.58 (m, 8H, 4 × CH_2_, H-3′, H-4′′, H-7′′, H-9′′), 1.80 (m, 4H, 2 × CH_2_, H-2, H-3), 1.98 (m, 3H, 3 × CH, H-3′′, H-5′′, H-8′′), 2.09 (m, 6H, 3 × CH_2_, H-2′′, H-6′′, H-10′′), 2.70 (t, 2H, CH_2_, H-1, *J* = 5.4 Hz), 2.93 (t, 2H, CH_2_, H-4, *J* = 5.7 Hz), 3.29 (m, 2H, CH_2_, H-5′), 3.59 (t, 2H, CH_2_, H-1′, *J* = 7.0 Hz), 3.88 (s, 3H, OCH_3_), 4.00 (m, 2H, 2 × CH, H-2′′′, H-3′′′), 6.63 (bs, 1H, NH), 6.89 (bs, 1H, NH), 7.37 (dd, 1H, CH, H-6, *J* = 9.2, 2.4 Hz), 7.55 (m, 1H, CH, H-8), 7.76 (d, 1H, CH, H-5, *J* = 9.2 Hz); ^13^C-NMR (DMSO-*d_6_*) *δ* 21.4, 22.3 (C-2, C-3), 23.9 (C-4′), 25.0 (C-1), 28.6 (C-3′′, C-5′′, C-8′′), 29.2 (C-4), 30.2 (C-3′), 30.4 (C-5′), 36.2 (C-4′′, C-7′′ , C-9′′), 41.4 (C-2′′, C-6′′, C-10′′), 42.9 (C-2′), 47.3 (C-1′), 52.7 (C-1′′), 55.9 (OCH_3_), 71.8 (C-2′′′, C-3′′′), 102.9 (C-8), 113.8 (C-9a), 119.0 (C-8a), 122.4 (C-6), 124.8 (C-5), 137.0 (C-10a), 152.3 (C-4a), 152.8 (C-9), 156.2 (C-7), 174.3 (C-1′′′, C-4′′′), 180.8 (C=S); Elemental analysis: calculated 62.17% C, 7.37% H, 8.53% N, 4.88% S; found 61.95% C, 7.52% H, 8.43% N, 5.02% S; ESI-MS: *m/z* 507.2 [M]^+^ (calculated for: [C_29_H_43_N_4_OS]^+^ 479.3).

*1-Adamantyl-3-(2-(7-methoxy-1,2,3,4-tetrahydroacridin-9-yl-amino)hexyl)thiourea 2,3-dihydroxy-succinate* (**15**). White-yellow powder, yield: 66.1%; m.p. = 91.8–94.4 °C; ^1^H-NMR (DMSO-*d_6_*) *δ* 1.25 (m, 4H, 2 × CH_2_, H-3′, H-4′), 1.38 (m, 2H, CH_2_, H-2′), 1.58 (m, 8H, 4 × CH_2_, H-5′, H-4′′, H-7′′, H-9′′), 1.79 (m, 4H, 2 × CH_2_, H-2, H-3), 1.98 (m, 3H, 3 × CH, H-3′′, H-5′′, H-8′′), 2.10 (m, 6H, 3 × CH_2_, H-2′′, H-6′′, H-10′′), 2.70 (t, 2H, CH_2_, H-1, *J* = 5.4 Hz), 2.93 (t, 2H, CH_2_, H-4, *J* = 5.6 Hz), 3.27 (m, 2H, CH_2_, H-6′), 3.56 (t, 2H, CH_2_, H-1′, *J* = 7.0 Hz), 3.88 (s, 3H, OCH_3_), 3.98 (m, 2H, 2 × CH, H-2′′′, H-3′′′), 6.51 (bs, 1H, NH), 6.87 (bs, 1H, NH), 7.35 (dd, 1H, CH, H-6, *J* = 9.2, 2.4 Hz), 7.53 (m, 1H, CH, H-8), 7.74 (d, 1H, CH, H-5, *J* = 9.2 Hz); ^13^C-NMR (DMSO-*d_6_*) *δ* 21.5, 22.4 (C-2, C-3), 25.1 (C-4′), 26.2 (C-1), 26.3 (C-6′), 28.8 (C-3′′, C-5′′, C-8′′), 29.2 (C-4), 30.5 (C-3′), 30.6 (C-5′), 36.2 (C-4′′, C-7′′, C-9′′), 41.4 (C-2′′, C-6′′, C-10′′), 42.9 (C-2′), 47.3 (C-1′), 52.7 (C-1′′), 55.8 (OCH_3_), 71.8 (C-2′′′, C-3′′′), 102.8 (C-8), 114.1 (C-9a), 119.2 (C-8a), 122.1 (C-6), 125.3 (C-5), 137.5 (C-10a), 152.5 (C-4a), 152.6 (C-9), 156.1 (C-7), 174.3 (C-1′′′, C-4′′′),180.8 (C=S); Elemental analysis: calculated 62.66% C, 7.51% H, 8.35% N, 4.78% S; found 62.40% C, 7.62% H, 8.50% N, 4.90% S; ESI-MS: *m/z* 521.2 [M]^+^ (calculated for: [C_31_H_45_N_4_OS]^+^ 521.3).

*1-Adamantyl-3-(2-(7-methoxy-1,2,3,4-tetrahydroacridin-9-yl-amino)heptyl)thiourea 2,3-dihydroxy- succinate* (**16**). White-yellow powder, yield: 74.5%; m.p. = 100.1–102.9 °C; ^1^H-NMR (DMSO-*d_6_*) *δ* 1.27 (m, 6H, 3 × CH_2_, H-3′, H-6′, H-7′), 1.38 (m, 2H, CH_2_, H-4′), 1.58 (m, 8H, 4 × CH_2_, H-5′, H-4′′, H-7′′, H-9′′), 1.80 (m, 4H, 2 × CH_2_, H-2, H-3), 1.98 (m, 3H, 3 × CH, H-3′′, H-5′′, H-8′′), 2.10 (m, 6H, 3 × CH_2_, H-2′′, H-6′′, H-10′′), 2.69 (t, 2H, CH_2_, H-1, *J* = 5.6 Hz), 2.94 (t, 2H, CH_2_, H-4, *J* = 5.7 Hz), 3.23 (m, 2H, CH_2_, H-7′), 3.58 (t, 2H, CH_2_, H-1′, *J* = 7.0 Hz), 3.88 (s, 3H, OCH_3_), 3.98 (m, 2H, 2 × CH, H-2′′′, H-3′′′), 6.52 (bs, 1H, NH), 6.88 (bs, 1H, NH), 7.35 (dd, 1H, CH, H-6, *J* = 9.2, 2.5 Hz), 7.54 (m, 1H, CH, H-8), 7.74 (d, 1H, CH, H-5, *J* = 9.2 Hz); ^13^C-NMR (DMSO-*d_6_*) *δ* 21.8, 22.7 (C-2, C-3), 25.4 (C-4′), 26.7 (C-1), 26.8 (C-6′), 29.0 (C-3′′, C-5′′, C-8′′), 29.1 (C-4), 29.5 (C-7′), 30.8 (C-3′), 30.9 (C-5′), 36.4 (C-4′′, C-7′′, C-9′′), 41.7 (C-2′′, C-6′′, C-10′′), 43.3 (C-2′), 47.6 (C-1′), 53.0 (C-1′′), 56.1 (OCH_3_), 72.0 (C-2′′′, C-3′′′), 103.1 (C-8), 114.3 (C-9a), 119.5 (C-8a), 122.5 (C-6), 125.6 (C-5), 137.8 (C-10a), 152.8 (C-4a), 152.9 (C-9), 156.4 (C-7), 174.6 (C-1′′′, C-4′′′), 181.1 (C=S); Elemental analysis: calculated 63.13% C, 7.65% H, 8.18% N, 4.68% S; found 63.40% C, 7.38% H, 8.01% N, 4.75% S; ESI-MS: *m/z* 535.2 [M]^+^ (calculated for: [C_32_H_47_N_4_OS]^+^ 535.3).

*1-Adamantyl-3-(2-(7-methoxy-1,2,3,4-tetrahydroacridin-9-yl-amino)octyl)thiourea 2,3-dihydroxy- succinate* (**17**). White-yellow powder, yield: 84.0%; m.p. = 75.1–77.8 °C; ^1^H-NMR (DMSO-*d_6_*) *δ* 1.24 (m, 8H, 4 × CH_2_, H-2′, H-3′, H-4′, H-6′), 1.40 (m, 2H, CH_2_, H-7′), 1.62 (m, 8H, 4 × CH_2_, H-5′, H-4′′, H-7′′, H-9′′), 1.82 (m, 4H, 2 × CH_2_, H-2, H-3), 2.00 (m, 3H, 3 × CH, H-3′′, H-5′′, H-8′′), 2.13 (m, 6H, 3 × CH_2_, H-2′′, H-6′′, H-10′′), 2.71 (m, 2H, CH_2_, H-1), 2.97 (m, 2H, CH_2_, H-4), 3.28 (m, 2H, CH_2_, H-8′), 3.68 (m, 2H, CH_2_, H-1′), 3.90 (s, 3H, OCH_3_), 4.07 (m, 2H, 2 × CH, H-2′′′, H-3′′′), 6.91 (bs, 1H, NH), 7.43 (dd, 1H, CH, H-6, *J* = 9.2, 2.4 Hz), 7.61 (m, 1H, CH, H-8), 7.79 (d, 1H, CH, H-5, *J* = 9.2 Hz); ^13^C-NMR (DMSO-*d_6_*): 21.2, 22.2 (C-2, C-3), 25.0 (C-1), 26.3, 26.5 (C-4′, C-5′), 28.8 (C-3′, C-6′, C-7′), 29.2 (C-3′′, C-5′′, C-8′′), 29.6 (C-4), 30.6 (C-2′), 36.2 (C-4′′, C-7′′, C-9′′), 41.4 (C-2′′, C-6′′, C-10′′), 43.0 (C-8′), 47.2 (C-1′), 52.7 (C-1′′), 55.9 (OCH_3_), 71.9 (C-2′′′, C-3′′′), 103.2 (C-8), 113.1 (C-9a), 118.5 (C-8a), 122.9 (C-6), 123.8 (C-5), 135.5 (C-10a), 151.6 (C-4a), 153.5 (C-9), 156.3 (C-7), 174.0 (C-1′′′, C-4′′′), 180.8 (C=S); Elemental analysis: calculated 63.58% C, 7.79% H, 8.02% N, 4.59% S; found 63.42% C, 7.62% H, 7.94% N, 4.70% S; ESI-MS: *m/z* 549.3 [M]^+^ (calculated for: [C_32_H_47_N_4_OS]^+^ 549.4).

### 3.5. Molecular Docking

Molecular modelling calculations were performed using AutoDock Vina [62]. The molecular models were built and minimized with UCSF chimera 1.3 (Amber Force Filed) [63]. The structure of both enzymes, human AChE (hAChE, PDB ID: 1B41) and human BChE (hBChE, PDB ID: 1P0I) were prepared using Pymol 1.1 from the crystal structures [64,65]. Compounds used in this study and both enzymes were prepared using AutoDock Tools 1.5.2. in charged form [56]. Molecules of water with other nonenzymatic molecules were removed (removing the fasciculin 2 from hAChE and molecules of water from both enzymes) and hydrogens were added. The 3D affinity grid box in the x-, y- and z- axes were 66, 66 and 66 with spacing 0.253 Å for hAChE, within the hBChE grid box dimensions were set to x = 46, y = 60, z = 46 with spacing 0.375 Å. For the hAChE docking the grid for energy was set in the coordinates x = 119.775, y = 117.597 and z = −128.964, within hBChE the coordinates were adjusted to x = 137.871, y = 115.156 and z = 38.652. The hAChE residues Trp86, Tyr72, Trp286, Asp74, Tyr341 and Phe297 were set to be flexible by AutoDock Tools 1.5.2, for hBChE amino-acid residues Glu325, His438, Trp82, Asp70 and Tyr332 were selected as flexible. Flexible ligand docking was performed for the selected compound **14** and reference compounds (THA, 7-MEOTA). The docking calculations were made on a Mac Pro 4.1 Quad-Core Intel Xeon 2.93 GHz system. At the end of the calculations, AutoDock Vina was used to perform cluster analysis. The visualization was carried out in Pymol 1.1. Hydrogens were finally removed to improve figure clarity.

## 4. Conclusions

In summary, a series of dual binding site cholinesterase inhibitors was designed and investigated. This new class of 7-MEOTA-adamantyl amine heterodimers with different linker sizes was prepared and tested for their ability to inhibit both targeted cholinesterases that are involved in proposed cholinergic hypothesis of AD. As shown in Table 1, all new compounds have good inhibitory activity to hBChE with IC_50_ values in the sub-μM range, and some of them also showed promising inhibitory activity towards hAChE. Our results highlighted compound **14** with a five methylene linker. This compound possessed the highest inhibitory activity for hAChE as well as for hBChE. Based on molecular modeling studies for molecule **14**, we have developed a novel agent that can directly interact with both the binding sites of hAChE. This study provided potentially important information for further development of THA- or 7-MEOTA-adamantyl amine analogues as valuable compounds for AD treatment. The inhibition of these series compounds to the channel activity of NMDA receptors will be tested.
